# Bat Coronavirus in Brazil Related to Appalachian Ridge and Porcine Epidemic Diarrhea Viruses

**DOI:** 10.3201/eid2104.141783

**Published:** 2015-04

**Authors:** Paulo Vitor Marques Simas, Ana Caroline de Souza Barnabé, Ricardo Durães-Carvalho, Daniel Ferreira de Lima Neto, Leonardo Cardia Caserta, Luiza Artacho, Fábio André Facco Jacomassa, Matheus Cavalheiro Martini, Márcia Mercês Aparecida Bianchi dos Santos, Paulo Anselmo Nunes Felippe, Helena Lage Ferreira, Clarice Weis Arns

**Affiliations:** University of Campinas, Campinas, São Paulo, Brazil (P.V.M. Simas, A.C.S. Barnabé, R. Durães-Carvalho, D.F. Lima-Neto, L.C. Caserta, L. Artacho, M.C. Martini, P.A.N. Felippe);; São Paulo State University, Rio Claro, São Paulo (F.A.F. Jacomassa);; Federal University of Juiz de Fora, Juiz de Fora, Brazil (M.M.A. Bianchi dos Santos);; São Paulo University, Pirassununga, Brazil (H.L. Ferreira).

**Keywords:** Coronavirus, viruses, Appalachian Ridge virus, porcine epidemic diarrhea virus, PEDV, CoV, Brazil, bats, Tadarida brasiliensis

**To the Editor**: *Tadarida brasiliensis* (I. Geoffroy, 1824) is a species of free-tailed bat that has resident and migratory populations in Brazil (*1*). This species has adapted to urban areas, occupying roofs, ceilings, and other human constructions, and often coexists with other bat species and humans (*2*), enabling epidemiologic risks (*3*). In recent studies, an alphacoronavirus has been detected in urban bat species *Molossus molossus*, *M. rufus,* and *Tadarida brasiliensi* in Brazil (*4*,*5*). Evidence suggests that alphacoronaviruses may use bats as hosts to spread human coronavirus (HCoV) NL63, which originated by evolution of Appalachian Ridge CoV strain 2 (ARCoV.2) (*6*).

In this study, a total of 20 anal and tracheal swab samples from 10 bats (*T. brasiliensis*) were collected at the Jequitibás Wood, in Campinas, São Paulo State, Brazil (22°54′31.34′′S 47°02′58.01′′W). We extracted viral genetic material using the RNA Extraction Mini Kit (QIAGEN, Hilden, Germany) and synthesized cDNA using random primers from the High Capacity cDNA Reverse Transcription Kit (Applied Biosystems, Foster City, CA, USA), following the manufacturer’s protocol.

Samples were analyzed by conventional reverse transcription PCR assays using panCoV primers targeting a 215-bp replicase fragment as previously described (*7*) with slight modifications to include more cycles and less extension time in order to obtain more PCR products. Sequencing reactions on a Pancoronavirus-positive anal swab sample (*7*) were performed at Central Laboratory of High Performance Technologies in Life Sciences (LaCTAD) at UNICAMP (http://www.lactad.unicamp.br) using an automated sequencer (3730xl DNA Analyzer; Applied Biosystems).

The chromatograms were edited using the program UGENE version 1.14 (UGENE, http://ugene.unipro.ru/forum/YaBB.pl?num=1407749393) and evaluated using Phred scores for base calling. Alignment was made with ClustalW v.2.1 software (http://www.clustal.org) implemented on Linux command line interface, and a similarity matrix was generated with sequences retrieved from the GenBank database. A 144-nt fragment of the replicase gene was obtained after editing, and phylogenetic analysis was performed after determining the best evolution model by using the jModelTest2 software (https://code.google.com/p/jmodeltest2/). Different CoV sequences were included to represent the genera *Alpha-, Beta-, Gamma-*, and *Deltacoronavirus*. Clustering with the ARCoV.2 and porcine epidemic diarrhea virus (PEDV) was obtained using the maximum-likelihood (ML) method after 1,000 Shimodaira-Hasegawa–like support values with the general time-reversible model and category approximation in 20 rates category in a gamma distribution (Technical Appendix Figure, panel A) and neighbor-joining methods under Kimura-2-parameter and 1,000 replicates of bootstrap (Technical Appendix Figure, panel B).

Subsequently, metagenomic analysis was made by creating a pool of the 10 bat samples. Samples were resuspended in Dulbecco Modified Eagle Medium (Life Technologies-GIBCO, Grand Island, NY, USA) and filtered through 0.22 μm. The recovered sample was then treated with DNase (Invitrogen, Carlsbad, CA, USA) to remove contaminating DNA and with Proteinase K (Invitrogen) to eliminate inhibitors and to disrupt viral capsids. Samples were then subjected to RNA extraction (QIAGEN) and sent to the sequencing core facility. Sequencing was performed on Illumina HiSeq2500 instrument by using the 2×100 bp kit according to manufacturer’s instructions. 

Through these analyses, we obtained 34,409,110 reads, of which 76.47% had quality index ≥30. The contigs were assembled by de novo genome assembly (blastx E-value ≤1^–5^) (*8*) generating 10.742 scaffolds: 35 matches for coronaviruses (using the Coronavirus Database, http://covdb.microbiology.hku.hk), 3 matches for PEDV, and 2 matches for HCoV-NL63 (both using the UniProt database, http://www.uniprot.org) (Technical Appendix). The sequences obtained had 87.5% (126/144) nucleotide identity with ARCoV.2, an unclassified alphacoronavirus (GenBank accession no. JX537912) for which a zoonotic role has been suggested (*6*). Preliminary analysis indicated good coverage of the polymerase region of the ARCoV.2 reference sequence by the reads (quality index >40) by using reference assembly against CoV complete genomes. This finding reinforces the hypothesis of this viral agent in the specimens analyzed. Moreover, molecular assays are underway in our laboratory to elucidate the alternative hypothesis of PEDV presence in bats in Brazil.

In summary, we found that a CoV detected in *T. brasiliensis* bats in Brazil has close phylogenetic relationships to ARCoV.2 and PEDV. Considering the zoonotic impact of these viral agents on the emergence of new diseases in animal and human populations, we believe that both results may strongly contribute to a better understanding of the molecular eco-epidemiology of these alphacoronaviruses. The reconstruction of their evolutionary history to trace their occurrence in humans and in bat populations as well as in other animals is being conducted to clarify their evolutionary pathway.

Technical Appendix Maximum likelihood and neighbor-joining genetic sequences of coronaviruses detected in bats of *Tadarida brasiliensis* species.
