# Supplementary material for: Bat Coronavirus in Brazil Related to Appalachian Ridge and Porcine Epidemic Diarrhea Viruses
Source: Emerg Infect Dis. 2015 Apr;21(4):729–31. doi: 10.3201/eid2104.141783 (PMC4378475; doi:10.3201/eid2104.141783)
Supplement: Technical Appendix — Maximum likelihood and neighbor-joining genetic sequences of coronaviruses detected in bats of Tadarida brasiliensis species. [file 14-1783-Techapp-s1.pdf]

Article DOI: http://dx.doi.org/10.3201/eid2104.121783

Coronavirus in Bats shows Relationship to Appalachian Ridge and Porcine Epidemic Diarrhea Viruses, Brazil

Technical Appendix

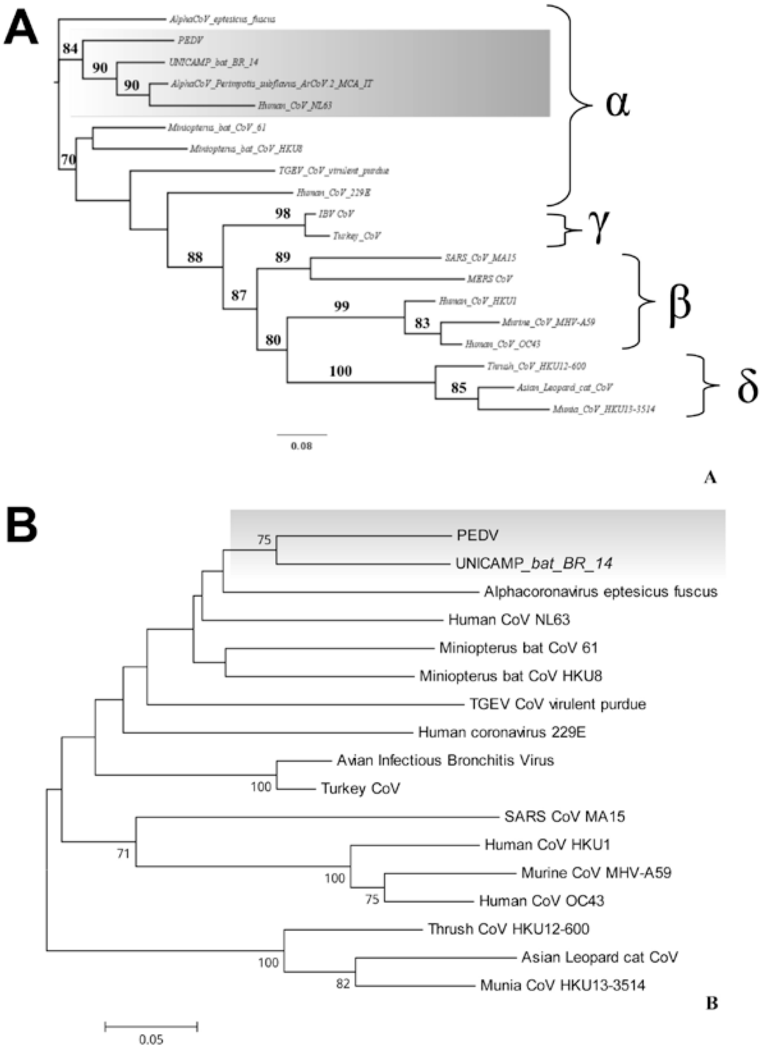

Figure: Phylogenetic trees obtained with the Maximum likelihood (using FastTree 2.1.7 software) A) and Neighbor-Joining (using MEGA v6.0) B) methods for the partial RdRp gene (144 bp). The sequences of this study are highlighted in blue with the name *UNICAMP_bat_BR_14* (submitted to GenBank under the

code KM514667). The GenBank no. accession sequences retrieved are described in order as shown in the trees: *AlphaCoV Eptesicus fuscus* (JX537914.1), PEDV (*Porcine Epidemic Diarrhea Coronavirus*, AF353511); UNICAMP_bat_BR_14; *AlphaCoV Perimyotis subflavus ARCoV.2 MCA IT* (JX537913.1); *Human CoV NL63* (AY567487); *Miniopterus bat CoV 61* (AY864196); *Miniopterus bat CoV HKU8* (DQ249228); *TGEV CoV virulent purdue* (*Transmissible Gastroenteritis Coronavirus*, DQ811789); *Human CoV 229E* (AF304460); *IBV* (*Avian Infectious Bronchitis Virus*, FJ904722); *Turkey CoV* (EU095850); *SARS CoV* (*Human Severe Acute Respiratory Syndrome CoV*, JF292915); *MERS CoV* (*Middle East Respiratory Syndrome*, KJ741000.1); *Human CoV HKU1* (AY597011); *Murine CoV MHV-A59* (FJ647225); *Human CoV OC43* (AY903460); *Thrush CoV HKU12-600* (FJ376621); *Asian Leopard cat CoV* (EF584908); *Munia CoV HKU13-3514* (FJ376622). No. branches represent the support values. Scale bars indicate no. nuclear substitutions per site.
